# A thermally activated delayed fluorescence exciplex to achieve highly efficient and stable blue and green phosphorescent organic light-emitting diodes†

**DOI:** 10.1039/c9ra02875g

**Published:** 2019-07-31

**Authors:** Dehai Dou, Peng Wu, Zhangcheng Liao, Jian Hao, Jianhua Zhang, Zixing Wang

**Affiliations:** Key Laboratory of Advanced Display and System Applications, Ministry of Education, Shanghai University 149 Yanchang Rd 200072 P. R. China zxwang78@shu.edu.cn jhzhang@shu.edu.cn +86-21-56333362 +86-21-56333362; Department of Chemistry, Shanghai University 149 Yanchang Rd 200072 P. R. China

## Abstract

The development of a thermally activated delayed fluorescence (TADF) exciplex with high energy is of great significance in achieving highly efficient blue, green, and red organic light-emitting diodes (OLEDs) for use in full-color displays and white lighting. Highly efficient and stable blue and green phosphorescent OLEDs were demonstrated by employing a TADF exciplex (energy: 2.9 eV) based on 4-substituted aza-9,9′-spirobifluorenes (aza-SBFs). Blue PhOLEDs demonstrated a maximum current efficiency (CE) of 47.9 cd A^−1^ and an external quantum efficiency (EQE) of 22.5% at 1300 cd m^−2^ (2.5 times the values of aza-SBF-based systems), with the best blue PhOLED demonstrating a CE, power efficiency (PE) and EQE of 60.3 cd A^−1^, 52.7 lm W^−1^, and 26.2%, respectively. Green PhOLEDs exhibited a CE of 78.1 cd A^−1^ and EQE of 22.5% at 9360 cd m^−2^, with the best green PhOLED exhibiting a maximum CE, PE, and EQE of 87.4 cd A^−1^, 101.6 lm W^−1^, and 24.5%, respectively. The device operational lifetime was improved over 17-fold compared to reference devices because of the high thermal stability of the materials and full utilization of the TADF exciplex energy, indicating their potential for application in commercial OLEDs.

## Abstract 2

A high energy TADF exciplex (415 nm) based on aza-spirobifluorene derivatives was demonstrated to achieve efficient and stable PhOLEDs.

## Graphical abstract

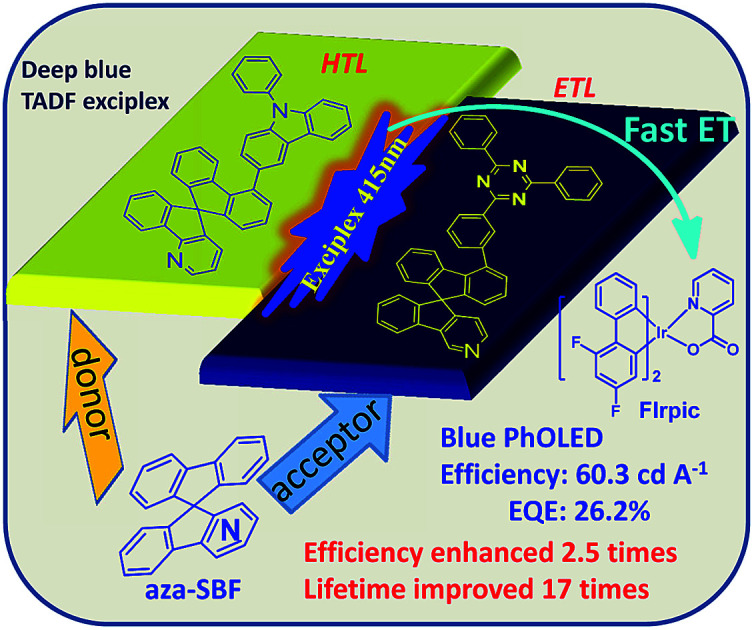

## Introduction

Since 1987, organic light-emitting diodes (OLEDs) have attracted intense interest because of their excellent applications in flat-panel displays and solid-state lighting.^1^ As a commercial requirement, the development of blue hosts and emitters has generated great interest in order to improve the efficiencies and operating lifetime as well as reduce the power consumption of OLEDs. 9,9′-Spirobifluorene (SBF)-based compounds,^2–10^ especially 4-substituted spiro-systems,^11–17^ have shown excellent performances for application in phosphorescent OLEDs (PhOLEDs), owing to their good solubilities, high triplet energy (*T*_1_), and good thermal and morphological stabilities. The introduction of electronegative nitrogen atoms to form bipolar materials could well balance electrons and holes in the emitting layer (EML) to achieve highly efficient OLEDs.^18–24^ Also, new analogues of SBFs, aza-9,9′-spirobifluorenes (aza-SBFs), have been developed by our group and highly efficient blue OLEDs have been demonstrated employing them as hosts.^25^ However, these aza-SBFs show deep highest occupied molecular orbitals (HOMOs) and high lowest unoccupied molecular orbitals (LUMOs), which are not able to inject holes and electrons smoothly, resulting in high operation voltage and fast efficiency roll-off. Meanwhile, they also exhibit high crystallinity without any glass transition temperature (*T*_g_), leading to low thermal stability under operation. Thus, the design of new aza-SBFs has become an interesting improvement by which to develop new materials systems with better charge mobility, higher *T*_g_, and suitable *T*_1_ and to then achieve highly efficient OLEDs with low roll-off.^26–30^ Indeed, the creation of aza-SBFs with substitutions at 4-position of the fluorene moiety leads to efficient π-conjugation breaking down due to strong steric congestion. It is known that a decrease in the LUMO energy levels through the incorporation of electron accepting moieties such as pyridine,^14,31,32^ triazine,^10,33–37^ or diphenylphosphine oxide,^38^ and an increase in the HOMO energy levels *via* the introduction of electron-donating groups such as arylamines,^39,40^ have remarkable consequences on the device performances. Highly efficient blue OLEDs have been demonstrated, through the development of new bipolar materials,^41,42^ novel hole or electron transport materials for dual EMLs^43^ and mixed hosts systems.^44–46^ Exciplexes formed between the electron transporting layer (ETL)/EML, hole transporting layer (HTL)/EML or HTL/ETL interfaces usually exhibit bipolar and thermally activated delayed fluorescence (TADF) as ideal hosts for PhOLEDs. The application of exciplex energy has been used to achieve high efficiencies, low turn-on voltage, and low roll-off.^43–52^

**Fig. 1:**
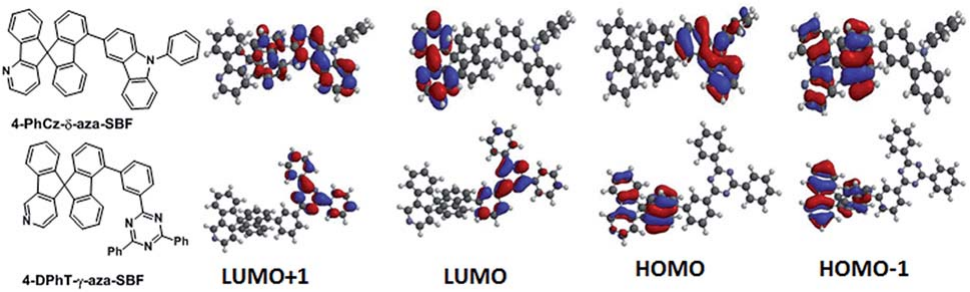
Chemical structures and electron density distributions of 4-PhCz-δ-aza-SBF and 4-DPhT-γ-aza-SBF.

Herein, the physical and electroluminescence (EL) properties of 4-substituted aza-SBFs with electron-withdrawing and -donating groups, respectively, were studied, as shown in Fig. 1. 4-(9-Phenyl-9*H*-carbazol-3-yl)-δ-aza-SBF (4-PhCz-δ-aza-SBF) shows high *T*_1_ (over 2.8 eV) and *T*_g_ (higher than 150 °C) values, and its HOMO energy levels (at −5.73 eV) are obviously higher than those of aza-SBFs (at −5.9 eV), which is of benefit to hole injection and transport. 4-(3-(4,6-Diphenyl-1,3,5-triazin-2-yl)phenyl)-γ-aza-SBF (4-DPhT-γ-aza-SBF) also exhibits high *T*_1_ (over 2.8 eV) and *T*_g_ (above 140 °C) values, while its LUMO energy levels (at −2.83 eV) are significantly lower than those of aza-SBFs (at −2.0 eV), meaning that it has excellent electron transporting capabilities. In particular, a deep blue TADF exciplex (energy ∼ 2.9 eV) was formed between 4-PhCz-δ-aza-SBF as a HTL and 4-DPhT-γ-aza-SBF as an ETL. Fully utilizing the singlet and triplet excitons, blue PhOLEDs based on a 4-PhCz-δ-aza-SBF/4-DPhT-γ-aza-SBF exciplex as a host demonstrated a maximum current efficiency (CE) of 60.3 cd A^−1^, a power efficiency (PE) of 52.7 lm W^−1^, and external quantum efficiency (EQE) of 26.2%. Blue PhOLEDs demonstrated a CE of 47.9 cd A^−1^ and EQE of 22.5% at 1300 cd m^−2^ (2.5 times the values of aza-SBF-based ones), and green PhOLEDs exhibited a CE of 78.1 cd A^−1^ and EQE of 22.5% at 9360 cd m^−2^, with the best green PhOLED exhibiting a maximum CE of 87.4 cd A^−1^, PE of 101.6 lm W^−1^, and EQE of 24.5%. The blue OLEDs show a lifetime of approximately 590 min for up to 90% (LT_(90)_) of the initial luminance of 2000 cd m^−2^ under a nitrogen atmosphere without encapsulation. The green OLEDs exhibit a lifetime of LT_(90)_ = 680 min for the initial luminance of 4500 cd m^−2^, which was very close to the TADF-based green OLEDs (LT_(90)_ = 700 min) in a previous report.^53^ With further device structural modification using suitable materials, stable PhOLEDs with much higher efficient could be achieved.

## Experimental

### General and photophysical measurements

All chemicals and reagents were used as received from commercial sources without further purification, unless stated otherwise. The auxiliary materials for OLED fabrication such as 1,4,5,8,9,11-hexaaza triphenylenehexacarbonitrile (HATCN), *N*,*N*′-dicarbazolyl-3,5-benzene (mCP), 4,4′-(cyclohexane-1,1-diyl)bis(*N*-phenyl-*N-p*-tolylaniline) (TAPC), 4,44′,4′′-tris-(*N*-carbazolyl)-triphenylamine (TCTA), *N*,*N*′-bis(1-naphthyl)-*N*,*N*′-diphenyl-1,1′-biphenyl-4,4′-diamine (NPB), 3,3′-(5′-(3-(pyridin-3-yl)phenyl)-1,1′:3′,1′′-terphenyl-3,3′′-diyl)dipyridine (TmPyPB), iridium(iii) bis(4,6-difluorophenyl)-pyridinato-N,C2′ picolinate (FIrpic), bis(2-phenylpyridine)iridium(iii) acetylacetonate (Ir(ppy)_2_acac), and lithium quinolin-8-olate (Liq) were purchased from Yurui (Shanghai) Chemical Co. Ltd. ^1^H NMR and ^13^C NMR spectra were recorded on a Bruker AV-500 spectrometer at room temperature. High-resolution mass spectra (HRMS) were determined on a Thermo Fisher Scientific LTQ FT Ultra mass spectrometer. Ultraviolet-visible (UV-vis) absorption spectra were recorded on a UV-2501PC instrument. Photoluminescence (PL) spectra were recorded using an FLSP920 fluorescence spectrophotometer, both in solution and solid state. Cyclic voltammetry (CV) and differential pulse voltammetry (DPV) were carried out using a CH Instrument 660C electrochemical analyzer with a Hg/Hg_2_Cl_2_ electrode as a reference electrode and tetra(*n*-butyl)ammonium hexafluorophosphate (TBAPF_6_) in dimethylformamide (DMF) as a supporting electrolyte. The *T*_g_ values of the compounds were determined under a nitrogen atmosphere by differential scanning calorimetry (DSC) on a TA Q500 HiRes thermal analyzer using a scanning rate of 10 °C min^−1^ with nitrogen flushing. The decomposition temperature (*T*_d_) corresponding to 5% weight loss was conducted on a TA Q500 HiRes thermogravimetric analysis (TGA) thermal analyzer.

### Device fabrication

The devices were fabricated by conventional vacuum deposition of the organic layer and cathode onto an indium tin oxide (ITO) coated glass substrate under a base pressure of lower than 1.0 × 10^−5^ Pa. All of the organic layers and aluminum cathode were subsequently deposited on pre-patterned glass substrates (26 × 31 mm) containing three ITO pixels (2 × 2 mm), which had been cleaned using scouring powder, acetone, and isopropyl alcohol followed by UV ozone treatment for 20 minutes. All of the organic layers and the Al cathode were deposited without exposure to the atmosphere. The deposition rates for the organic materials and Al were typically 1.0 and 5.0 Å s^−1^, respectively.

### Device measurements

The current density–voltage–luminescence (*J*–*V*–*L*) characteristics were measured by a Keithley 2400 source meter and a PR-650 Spectra Colorimeter in the direction perpendicular to the substrate at room temperature under ambient conditions.

## Results and discussion

### Synthesis

**Scheme 1:**
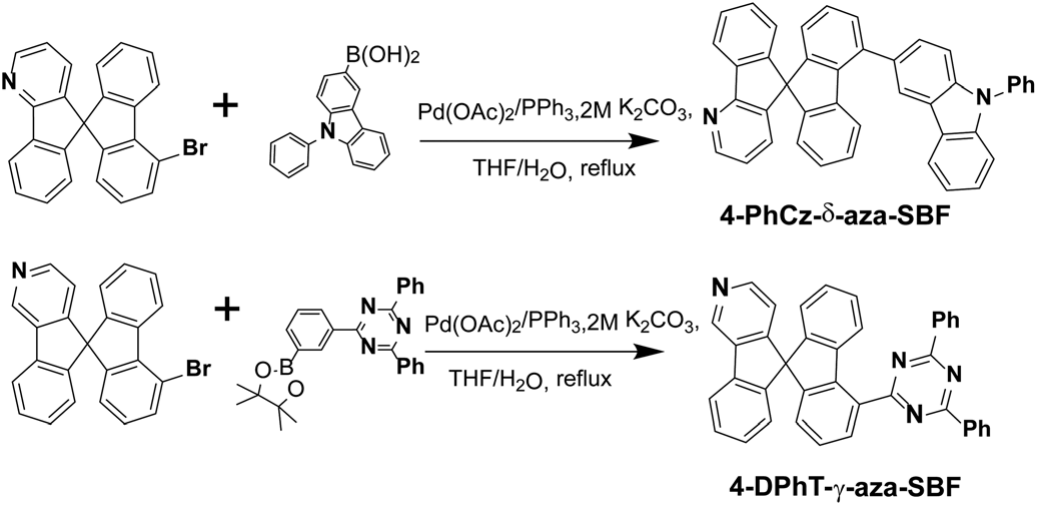
Synthetic routes for 4-PhCz-δ-aza-SBF and the 4-DPhT-γ-aza-SBF.

The synthetic routes of the 4-substituted aza-SBFs are shown in Scheme 1. 2,4-Diphenyl-6-(3-(4,4,5,5-tetramethyl-1,3,2-dioxaborolan-2-yl)phenyl)-1,3,5-triazine reacted with 4-bromo-γ-aza-SBF under general Suzuki coupling reaction conditions gave 4-DPhT-γ-aza-SBF in 87% yield. 9-Phenyl-9*H*-carbazol-3-ylboronic acid also reacted with 4-bromo-δ-aza-SBF to obtain 4-PhCz-δ-aza-SBF in 91% yield. ^1^H and ^13^C NMR spectroscopy and HRMS were used to characterize the final compounds.

### Thermal properties

The thermal stabilities and morphological properties of the two compounds were estimated using TGA and DSC. The decomposition temperature (*T*_d_) corresponding to 5% weight loss was recorded at 377 °C for 4-PhCz-δ-aza-SBF and at 430 °C for 4-DPhT-γ-aza-SBF, respectively. These compounds appear to be more stable than their constituent building block aza-SBFs (*T*_d_: 252 °C).^25^ The constituent building block aza-SBFs show clear melting temperatures at around 234 °C, whereas high *T*_g_ values were also observed for these two materials without any melting occurring during the DCS measurements. 4-PhCz-δ-aza-SBF showed *T*_g_ at 153 °C and 4-DPhT-γ-aza-SBF exhibited *T*_g_ at 146 °C (Fig. S1† and Table 1). As observed above for the *T*_g_ values, these materials showed significantly higher *T*_g_ values than 4-PhCz-SBF (*T*_g_: 127 °C)^13^ and also higher than those of most SBF-based materials with similar substituents. With such high *T*_d_ and *T*_g_ values, we believe that these materials do not undergo real decomposition when employed as functional materials during device fabrication under high vacuum.

**Table 1:** Photophysical, electrochemical, and thermal properties of 4-PhCz-δ-aza-SBF and 4-DPhT-γ-aza-SBF

Compound	*λ*_abs_ [nm] Sol.^a^	*λ*_PL_ [nm] Sol.^a^	*λ*_Ph_ [nm] Sol.^b^	*E*_ox_/*E*_red_^c^ [V]	HOMO/LUMO [eV]	*E*_g_ f/*T*_1_^g^ [eV]	*T*_g_/*T*_d_^h^ [°C]
4-PhCz-δ-aza-SBF	313, 346	376, 388	436	0.93/−2.75	−5.74^d^/−2.29	3.46/2.84	153/377
4-DPhT-γ-aza-SBF	272, 306	391	441	na/−1.97	−6.71/−2.83^e^	3.88/2.81	146/430

a Measured in 2-methyl-THF solutions at room temperature.

b Measured in 2-methyl-THF solutions at 77 K.

c *E*_ox_ = oxidation potential and *E*_red_ = reduction potential.

d The HOMO value: *E*_HOMO_ (eV) = −(*E*_ox_ + 4.8).

e The LUMO value: *E*_LUMO_ (eV) = −(*E*_red_ + 4.8).

f The value of *E*_g_ was calculated from the absorption edges of the solutions.

g The value of *T*_1_ was estimated from the onset values of the phosphorescence spectra (*λ*_Ph_).

h Obtained from DSC and TGA measurements; na = none appears.

### Theoretical calculations

Density functional theory (DFT) calculations were performed using the most popular Gaussian 09 software, employing a B3LYP functional with the 6-31G* basis set. In the case of 4-PhCz-δ-aza-SBF, the HOMO is delocalized on the strong electron-donating unit of the 9-phenyl-9*H*-carbazole (PhCz).^13^ The dihedral angles are over 65° between the fluorene and carbazole units owing to strong steric congestion, which efficiently disrupted the π-conjugation between these two components, resulting in a very small influence on the fluorene moiety. The LUMO distribution of 4-PhCz-δ-aza-SBF was similar on the aza-fluorene moieties, irrespective of the position of nitrogen, owing to their strong electron deficient character. The LUMO+1 orbital is delocalized on the carbazole-fluorene units (Fig. 1). This orbital might play an important role in electron transitions. The HOMO of 4-DPhT-γ-aza-SBF not only localized on the fluorene unit, but also on the azafluorene component, which is very similar to the HOMO orbital distribution of aza-SBFs.^25^ The strong electron deficiency of the triazine moiety results in both the LUMO and LUMO+1 orbitals of 4-DPhT-γ-aza-SBF localizing on it. According to calculations, the triphenyl-1,3,5-triazine unit displays an almost planar configuration, with dihedral angles of close to 60° between the fluorene and triphenyl-1,3,5-triazine units.

### Electrochemical properties

The electrochemical properties of the two materials were examined by CV and DPV. The electrochemical reversibility was determined using CV (Fig. S2†), while all redox potentials were found using DPV and reported relative to a ferrocenium/ferrocene (Fc^+^/Fc) redox couple as an internal standard. The reverse oxidation progress of 4-PhCz-δ-aza-SBF was observed at 0.93 V, attributed to the PhCz moiety,^13^ which is consistent with the DFT calculations that the HOMO orbitals can be attributed to PhCz. The irreversible reduction potential of 4-PhCz-δ-aza-SBF was observed at −2.75 V, attributed to the azafluorene unit. There was no obvious oxidation observed for 4-DPhT-γ-aza-SBF because of the lack of an electron-donating moiety. On the contrary, its reduction behavior showed more information. The first reduction is at around −2.0 V, corresponding to the electron-deficient moiety of triazine. Since the azafluorene unit could not conjugate with the triazine part due to the sp^3^ hybridized carbon of the connection and large dihedral angle, they have much less interaction with each other. The second and third reductions that occur at around −2.83 V were assigned to the reduction of the azafluorene and fluorene units, similar to those of 4-PhCz-δ-aza-SBF.

### Photophysical properties

**Fig. 2:**
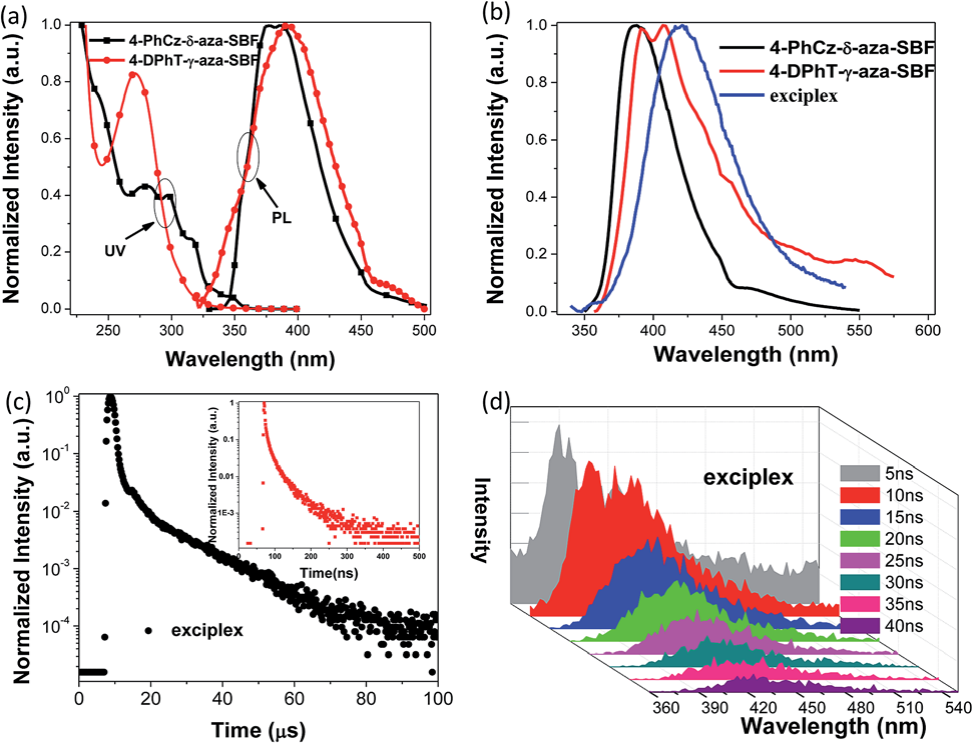
(a) UV-vis absorption (UV) and PL spectra of 4-PhCz-δ-aza-SBF and 4-DPhT-γ-aza-SBF in 2-MeTHF solutions, (b) PL spectra of 4-PhCz-δ-aza-SBF, 4-DPhT-γ-aza-SBF and co-deposited 4-PhCz-δ-aza-SBF : 4-DPhT-γ-aza-SBF (1 : 1) films (exciplex), (c) PL transient decay curves of the exciplex at 420 nm, inset: prompt fluorescence measured in the range of 0 to 500 ns, and (d) transient PL spectra of the exciplex delayed after 5, 10, 15, 20, 25, 30, 35, and 40 ns.

The UV-vis absorption spectra of the two materials recorded in 2-methyltetrahydrofuran (2-MeTHF) are presented in Fig. 2. 4-PhCz-δ-aza-SBF showed a weak absorption band with a maximum at 346 nm, due to n–π* transitions occurring on the PhCz fragment, leading to an optical energy gap (*E*_g_) from the onset of the absorption band at 3.46 eV.^13^ The stronger absorption bands with a maximum at around 310 nm are attributed to the n–π* transition of aza-SBFs moieties similar to those of the main absorption bands of aza-SBFs.^25^ The absorption in the 260–300 nm range might be assigned to the interaction as well as the sum spectra of the π–π* transition of these two components. 4-DPhT-γ-aza-SBF exhibited two clear absorption bands with maxima at 272 and 306 nm, attributed to phenyltriazine and aza-SBF units, respectively. On the basis of DFT calculations, the HOMOs of 4-DPhT-γ-aza-SBF were mainly localized on fluorene moieties, and the strong electron deficiency of triazine stabilized the HOMO energy levels. The *E*_g_ of 4-DPhT-γ-aza-SBF at 3.88 eV is slightly larger than that of 4-PhCz-δ-aza-SBF. Their HOMO and LUMO energy levels were determined from their first oxidation or reduction potential as well as with the assistance of *E*_g_, (HOMO = −(4.8 + *E*_ox_), LUMO = −(4.8 + *E*_red_), LUMO = HOMO + *E*_g_).^32^ The HOMO energy level of 4-PhCz-δ-aza-SBF at −5.74 eV is very close to that of the PhCz unit (−5.6 eV) confirming the key importance of the introduction of the PhCz unit to adjust the HOMO energy level compared with that of aza-SBF (at −5.9 eV).^25^ The electron deficient aza-SBF units slightly stabilize the HOMO orbitals due to incomplete conjugation between aza-SBF and the PhCz unit. On the other hand, the electron-donating ability of PhCz slightly affects the LUMO energy levels. However, the *E*_g_ values of 4-PhCz-δ-aza-SBF decreased significantly, resulting in deeper LUMO energy levels at −2.29 eV compared with those of aza-SBF (at −2.0 eV). The LUMO energy levels of 4-DPhT-γ-aza-SBF at −2.83 eV were estimated by their first reduction potentials since they did not show any oxidation potentials over the CV testing range.

The photoluminescence (PL) spectra of these 4-substituted aza-SBFs were recorded in a diluted solution of 2-MeTHF at room temperature and at 77 K respectively. 4-PhCz-δ-aza-SBF showed emission spectra with maximum peaks at 376 and 388 nm. 4-DPhT-γ-aza-SBF exhibited very weak emission at 395 nm in solution. The phosphorescent PL spectra of these two compounds were also recorded to estimate their triplet energy levels (*T*_1_ > 2.8 eV) (Fig. S3† and Table 1). 4-PhCz-δ-aza-SBF and 4-DPhT-γ-aza-SBF were co-deposited in a mole ratio of 1 : 1 to form a film. As shown in Fig. 2b, an extra emission peak at 418 nm was observed for the film (exciplex emission, 2.95 eV), which matches the energy difference between the LUMO of 4-DPhT-γ-aza-SBF (−2.83 eV) and the HOMO of 4-PhCz-δ-aza-SBF (−5.74 eV) with a PL quantum efficiency of 23.9%. Because of the high energy (2.95 eV) of the exciplex formed by the two materials, it is easy to consume heat energy or other forms of consumption, but it can also be consumed by oxygen, which leads to the low quantum yield of the exciplex. Transient PL spectra of the exciplex were also recorded with delay times of 5, 10, 15, 20, 25, 30, 35, and 40 ns, respectively (Fig. 2d). Both emissions of 4-PhCz-δ-aza-SBF and the exciplex were observed as initial excitations. However, only the exciplex emission remained when the spectra were recorded after a 15 ns delay. The transient decay curve at 418 nm can be resolved into two components: the prompt component and the delayed component were estimated to be 14.4 ns (28.5%) and 13.3 μs (71.5%), from second-order exponential decay fitting. On the basis of literature equations,^43^ the kinetic parameters of the co-deposited film were estimated: the prompt fluorescence decay rate constant (*k*_p_) at 6.94 × 10^7^ s^−1^, the delayed fluorescence decay rate constant (*k*_d_) at 7.52 × 10^4^ s^−1^, the rate constants of the ISC and RISC processes (*k*_ISC_ and *k*_RISC_) at 6.15 × 10^7^ and 1.67 × 10^5^ s^−1^, the radiative decay rate constant of the S_1_ state to the ground state (*k*^s^_r_) at 7.97 × 10^6^ s^−1^, and the non-radiative decay rate constant of the *T*_1_ state (*k*^T^_nr_) at 5.61 × 10^4^ s^−1^, respectively. The value of *k*^T^_nr_ was only 30% of that of *k*_RISC_, which means that triplet excitons prefer to up-convert to the singlet state and generate delayed fluorescence.

### Device performance

**Fig. 3:**
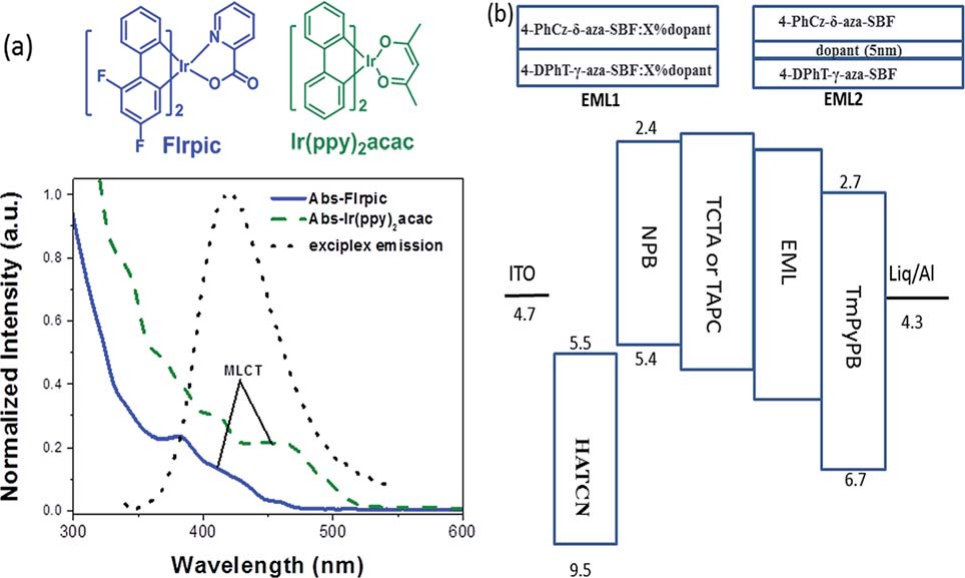
(a) Chemical structures and UV absorption spectra of FIrpic and Ir(ppy)_2_acac and the PL spectrum of the exciplex and (b) the device structure with EML1 and EML2.

The exciplex emission has very high energy as well as TADF characteristics, its emission spectrum matches the absorption spectra of most iridium complexes (Fig. 3). So, it could be an ideal host for phosphorescent emitters, through Förster resonance (FRET) and Dexter energy transfer. Conventional blue (FIrpic) and green (Ir(ppy)_2_acac) dopants were selected to fabricate the PhOLEDs. Two different emitting layers were designed to evaluate the energy transfer between the exciplex and dopants. The exciplex was formed at the interface between 4-PhCz-δ-aza-SBF and 4-DPhT-γ-aza-SBF, then a 5 nm neat film of FIrpic or Ir(ppy)_2_acac was inserted at the interface to form a non-doped EML (EML 1: 4-PhCz-δ-aza-SBF (20 nm)/dopant (5 nm)/4-DPhT-γ-aza-SBF (20 nm)). The exciplex could also diffuse into the 4-PhCz-δ-aza-SBF and 4-DPhT-γ-aza-SBF layers, so the phosphorescent emitters were doped into these two materials to form dual emitting layers (EML2: 4-PhCz-δ-aza-SBF : *x*% dopant (20 nm)/4-DPhT-γ-aza-SBF : *x*% dopant (20 nm)).

**Fig. 4:**
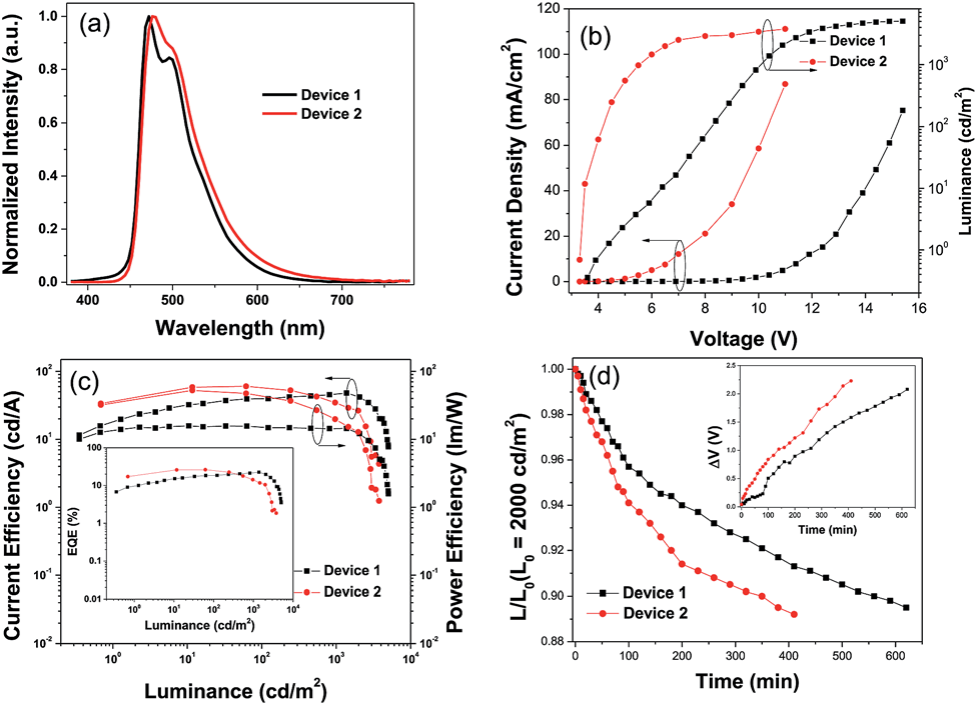
(a) EL spectra, (b) *J*–*V*–*L* characteristics, and (c) CE-L-PE curves of the blue PhOLED devices 1 and 2, inset: EQE-L curves, and (d) time evolution of the normalized luminance, *L*, of devices 1 and 2 under a nitrogen atmosphere (inset: change in the operating voltage Δ*V* (offset to zero)) at an initial luminance of *L*_0_ = 2000 cd m^−2^.

Blue PhOLEDs with ITO/HATCN (10 nm)/NPB (10 nm)/TCTA (10 nm)/4-PhCz-δ-aza-SBF : 6% FIrpic (20 nm)/4-DPhT-γ-aza-SBF : 6% FIrpic (20 nm)/TmPyPB (40 nm)/Liq (1 nm)/Al (80 nm) (device 1) and ITO/HATCN (10 nm)/NPB (10 nm)/TCTA (10 nm)/4-PhCz-δ-aza-SBF (20 nm)/FIrpic (5 nm)/4-DPhT-γ-aza-SBF (20 nm)/TmPyPB (40 nm)/Liq (1 nm)/Al (80 nm) (device 2) architectures were fabricated. The EL spectra and current density–voltage–luminance (*J*–*V*–*L*) characteristics of these PhOLEDs are exhibited in Fig. 4 and the devices performances are summarized in Table 2. As shown in Fig. 4a, these devices only exhibited typical emission spectra for FIrpic at 472 and 495 nm without any other peaks observed in the range of 380–460 nm, indicating full exciton energy utilization. The turn on voltage of device 1 was at 3.9 V, which was slightly lower than that of the aza-SBF-based blue OLEDs (∼4.1 V) due to the higher HOMO energy levels of 4-PhCz-δ-aza-SBF. Interestingly, the luminance of device 1 increased significantly, resulting in an increase in efficiency, demonstrating rare efficiency roll-off upon an increase in the operation voltage. Device 1 showed a maximum CE of 47.9 cd A^−1^ and EQE of 22.5% at 1300 cd m^−2^ (Fig. 4b and c). The energy difference between the LUMO levels of 4-PhCz-δ-aza-SBF and 4-DPhT-γ-aza-SBF are around 0.6 eV, and the differences in the HOMO levels are around 0.95 eV. So, holes will gather at the HOMO levels of 4-PhCz-δ-aza-SBF, while electrons prefer to stay at the LUMO levels of 4-DPhT-γ-aza-SBF. Because of the high energy barriers, the hole and electron could not combine, neither in the 4-PhCz-δ-aza-SBF layer nor in 4-DPhT-γ-aza-SBF. Therefore, an exciplex formed at the interface of the two. Rare exciplex formation leads to low luminance of the device at low operation voltage. As the driving voltage increased, more exciplexes occurred, meanwhile the energy barriers were overcome and contributed to more holes and electrons combining in the EML, resulting in an enhancement in the efficiency and luminance. In order to harvest exciplex energy efficiently, an ultrathin film of FIrpic was inserted between them. Device 2 demonstrated a maximum CE of 60.3 cd A^−1^, a PE of 52.7 lm W^−1^, and an EQE of 26.2% with lower turn on voltage at 3.4 V, which was more efficient than that of device 1 and very close to the world record at 62.2 cd A^−1^.^44^ The energy of the exciplex was harvested by FIrpic directly through FRET and Dexter energy transfer to achieve high efficiencies. Device 2 still retained 58.8 cd A^−1^ at 100 cd m^−2^, but decreased to 34.2 cd A^−1^ at 1000 cd m^−2^, the possible reason for this being the self-quenching of the triplet state of FIrpic.

**Table 2:** Performances of blue and green OLEDs based on 4-PhCz-aza-SBF and 4-DPhT-aza-SBF as a host

Device	*V*_on_ (V)	PE/CE/EQE [lm W^−1^/cd A^−1^/%]			CIE (*x*, *y*)
		Max	At 100 cd m^−2^	At 1000 cd m^−2^	
1	3.9	15.8/47.9/22.5	15.4/39.4/18.5	14.4/46.6/21.9	(0.164, 0.344)
2	3.4	52.7/60.3/26.2	45.1/58.8/25.2	19.4/34.2/14.1	(0.163, 0.332)
3	3.0	43.4/78.1/22.5	36.5/59.3/17.3	31.1/67.9/19.1	(0.320, 0.619)
4	2.3	101.6/87.4/24.6	90.1/80.7/22.8	59.2/67.4/19.0	(0.357, 0.610)
5	3.6	30.5/51.1/14.9	25.0/49/14.4	19.9/50.1/14.6	(0.319, 0.619)

The operation lifetimes of device 1 and 2 were measured under a nitrogen atmosphere without any encapsulation. Fig. 4d shows the time evolution of the luminance, *L*, and the change in the voltage from its initial value, Δ*V* = |*V*(*t* = 0) − *V*(*t*)|. Devices were operated at a constant current density (∼10 mA cm^−2^) to give an initial luminance of 2000 cd m^−2^ at room temperature. Device 1 exhibited a longer lifetime of approximately 560 min for up to 90% (LT_90_) of the initial luminance, which was 1.6 times that of device 2 (LT_90_ = 350 min) and over 20 times those of reference devices (such as an mCP-based blue PhOLED, with an LT_90_ of less than 20 min).

**Fig. 5:**
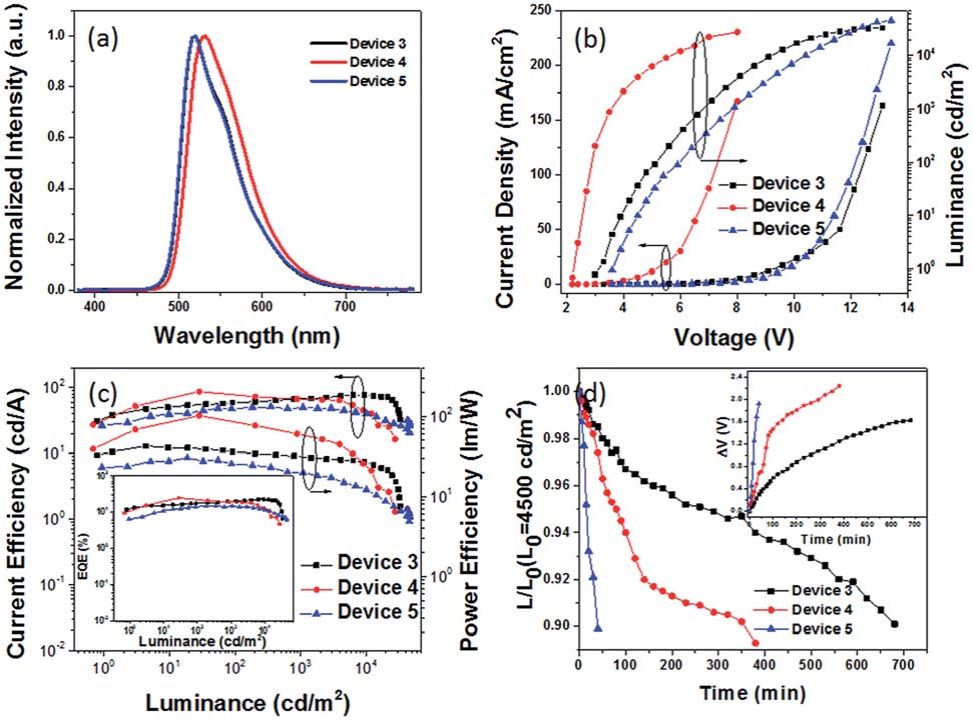
(a) EL spectra, (b) *J*–*V*–*L* characteristics, and (c) CE–L–PE curves of green PhOLED devices 3–5, where the inset shows the EQE–L curves, and (d) time evolution of the normalized luminance, *L*, of devices 3–5 under a nitrogen atmosphere (inset: change in the operating voltage Δ*V* (offset to zero)) at an initial luminance of *L*_0_ = 4500 cd m^−2^.

The hole and electron recombination zone was very close to the ETL due to the electron mobility being much lower than that of the holes in common OLEDs. On the basis of the blue PhOLEDs, a thicker TAPC (30 nm) was selected as both the hole transporting and electron blocking layer owing to its higher LUMO energy level (at 2.0 eV) than that of TCTA, and a thinner TmPyPB layer (30 nm) was deposited to enhance the electron transport and adjust the recombination zone. Then, green PhOLEDs with ITO/HATCN (10 nm)/NPB (5 nm)/TAPC (30 nm)/4-PhCz-δ-aza-SBF : 8% Ir(ppy)_2_acac (20 nm)/4-DPhT-γ-aza-SBF : 8% Ir(ppy)_2_acac (20 nm)/TmPyPB (30 nm)/Liq (1 nm)/Al (80 nm) (device 3) and ITO/HATCN (10 nm)/NPB (5 nm)/TAPC (30 nm)/4-PhCz-δ-aza-SBF (20 nm)/Ir(ppy)_2_acac (5 nm)/4-DPhT-γ-aza-SBF (20 nm)/TmPyPB (30 nm)/Liq (1 nm)/Al (80 nm) (device 4) architectures were also fabricated and characterized. The EL spectra and *J*–*V*–*L* characteristics of these PhOLEDs are exhibited in Fig. 5. The comparison device 5 (ITO/HATCN (10 nm)/NPB (5 nm)/TAPC (30 nm)/CBP : 8% Ir(ppy)_2_acac (20 nm)/TmPyPB (30 nm)/Liq (1 nm)/Al (80 nm)) was also constructed using common material CBP as a host. The EL spectra of the green OLEDs exhibit the emission of the green dopant (Ir(ppy)_2_acac) at 518 nm without any peaks observed in the 380–460 nm range (Fig. 5a). The turn on voltage of device 3 decreased to 3.0 V, and its maximum CE and EQE increased to 78.1 cd A^−1^ and 22.5% at very high luminance (at 9300 cd m^−2^) with hardly any roll-off. Compared with the performance of device 5 using CBP as a host (maximum CE of 50.1 cd A^−1^ and EQE of 14.9%, at 350 cd m^−2^), the efficiency of device 3 was enhanced by over 50%.

Similar to the blue PhOLEDs, non-doped green PhOLEDs (device 4) were also fabricated. Optimized device 4 (5 nm of Ir(ppy)_2_acac insert) displayed extremely high efficiencies with a maximum CE of 87.4 cd A^−1^, PE of 101.6 lm W^−1^, and EQE of 24.6% with a very low turn on voltage at 2.3 V, which is the same as its emission peak (532 nm) photon energy (2.3 eV). Thus, the operation lifetimes of devices 3, 4, and 5 were measured to study the stability of 4-PhCz-δ-aza-SBF and 4-DPhT-γ-aza-SBF as hosts under a nitrogen atmosphere without any encapsulation. Fig. 5d shows the time evolution of the luminance, *L*, and the change in voltage from their initial value, Δ*V* = |*V*(*t* = 0) – *V*(*t*)|. The devices were operated at a constant current density (∼10 mA cm^−2^) for an initial luminance of 4500 cd m^−2^ at room temperature. Surprisingly, device 3 exhibited a long lifetime of LT_90_ = 680 min, which was 17 times that of the reference device 5 (LT_90_ = 40 min). Device 3 showed even better performances than in our previous work using TADF materials as a host.^53^ Similar to the blue PhOLEDs, non-doped green device 4 showed a lower operation lifetime (LT_90_ = 350 min), which means that the lifetime of non-doped devices might be dependent on the thickness of the phosphorescent emitters, since the quenching mold is attributed to the triplet state of the phosphor. The high efficiency can be attributed to the exciplex formation between 4-PhCz-δ-aza-SBF and 4-DPhT-γ-aza-SBF. In the efficient reverse ISC from the triplet to the singlet of the exciplex, the singlet energy of the exciplex transfers very quickly to the dopant though FRET,^50,51,53^ reducing the triplet density on the host. In addition, charges are effectively confined in the EML due to a large energy barrier for hole and electron leakage, requiring hole and electron transport materials that show low degradation. Also, the high *T*_g_ values of the 4-substituted-aza-SBFs also have a positive effect on the lifetime to prevent the crystallization of the EML.

## Conclusions

In summary, a TADF exciplex with high energy (>2.9 eV) was achieved between 4-PhCz-δ-aza-SBF and 4-DPhT-γ-aza-SBF. Highly efficient and stable blue and green phosphorescent OLEDs were demonstrated by employing them as a host. Non-doped blue PhOLEDs demonstrated a maximum CE of 60.3 cd A^−1^, PE of 52.7 lm W^−1^, and EQE of 26.2%, and non-doped green PhOLEDs showed a maximum CE of 87.4 cd A^−1^, PE of 101.6 lm W^−1^, and EQE of 24.6% with a low turn-on voltage of 2.3 V. A stable blue PhOLED based on 4-PhCz-δ-aza-SBF : 6% Firpic (20 nm)/4-DPhT-γ-aza-SBF : 6% Firpic (20 nm) as an EML demonstrated a CE of 47.9 cd A^−1^ and an EQE of 22.5% at 1300 cd m^−2^ with LT_90_ = 560 min (at ∼10 mA cm^−2^). A stable green PhOLED based on 4-PhCz-δ-aza-SBFs : 8% Ir(ppy)_2_acac (20 nm)/4-DPhT-γ-aza-SBF : 8% Ir(ppy)_2_acac as an EML exhibited a CE of 78.1 cd A^−1^ and an EQE of 22.5% at 9360 cd m^−2^ with LT_90_ = 680 min, which was improved over 17-fold compared with the values of reference PhOLEDs. The high thermal stability of the 4-substituted aza-SBFs with a high *T*_1_, as well as the formation of a high energy TADF exciplex, make them ideal host materials for OLEDs with a long lifetime. Further device structural modification with suitable materials could result in stable OLEDs that show higher efficiency.

### Synthesis of the compounds

#### General procedure of the Suzuki-coupling reaction

4-Bromo-aza-SBF (2.0 g, 5 mmol), 2,4-diphenyl-6-(3-(4,4,5,5-tetramethyl-1,3,2-dioxaborolan-2-yl)phenyl)-1,3,5-triazine (2.5 g, 6 mmol) or 9-phenyl-9*H*-carbazol-3-ylboronic acid (1.6 g, 6 mmol), and potassium carbonate (2 g, 14 mmol) were dissolved in THF (100 mL) and water (40 mL). After 15 min of N_2_ bubbling, Pd(OAc)_2_ (0.1 g, 0.44 mmol) and PPh_3_ (0.4 g, 1.52 mmol) were added and then refluxed for 12 h under a N_2_ atmosphere. After cooling to room temperature, the mixture was poured into water and then extracted with dichloromethane. The organic phase was dried with anhydrous MgSO_4_ before being evaporated. The residue was purified by column chromatography on silica gel with CH_2_Cl_2_/ethyl acetate as an eluent to yield the desired product in over 80% yield. Sublimation purification was also undertaken to give the materials in high purity.

#### 4-(9-Phenyl-9*H*-carbazol-3-yl)spirofluorene-9,9′-indeno1,2-bpyridine (4-PhCz-δ-aza-SBF)

Yield: 71%.^1^H NMR (500 MHz, CDCl_3_) *δ* 8.57 (s, 1H), 8.34 (s, 1H), 8.15 (s, 1H), 7.90–7.75 (m, 1H), 7.74–7.57 (m, 3H), 7.57–7.50 (m, 3H), 7.49–7.35 (m, 4H), 7.33 (s, 1H), 7.27 (dd, *J* = 7.9, 8.1 Hz, 2H), 7.15 (m, 1H), 7.11 (dd, *J* = 8.6, 4.4 Hz, 2H), 7.07 (s, 1H), 7.00 (s, 1H), 6.95 (s, 1H), 6.87 (d, *J* = 7.3 Hz, 1H), 6.78–6.56 (m, 2H). ^13^C NMR (126 MHz, CDCl_3_) *δ* 160.83, 149.69, 149.01, 148.10, 147.73, 143.48, 142.03, 141.38, 140.48, 139.07, 138.80, 137.70, 132.56, 130.83, 130.02, 128.34, 127.64, 127.57, 127.46, 127.16, 126.29, 123.77, 123.62, 123.44, 123.22, 122.64, 122.21, 121.11, 120.54, 120.20, 110.05, 109.83. HRMS (ESI) calculated for C_42_H_26_N_2_ M + H^+^ 559.2162, found: 559.2169.

#### 4-(3-(4,6-Diphenyl-1,3,5-triazin-2-yl)phenyl)spirofluorene-9,9′-indeno1,2-cpyridine (4-DPhT-γ-aza-SBF)

Yield: 87%. ^1^H NMR (500 MHz, CDCl_3_) *δ* 9.16 (s, 1H), 9.00–8.93 (m, 2H), 8.81 (d, *J* = 7.1 Hz, 4H), 8.41 (d, *J* = 4.9 Hz, 1H), 7.97 (d, *J* = 8.0 Hz, 1H), 7.88 (d, *J* = 7.3 Hz, 1H), 7.67–7.61 (m, 3H), 7.59–7.53 (m, 3H), 7.45 (dd, *J* = 7.6 Hz, 2H), 7.38 (d, *J* = 7.4 Hz, 1H), 7.25–7.19 (m, 2H), 7.11 (d, *J* = 8.0 Hz, 1H), 7.08–7.01 (m, 2H), 6.91 (d, *J* = 7.5 Hz, 1H), 6.83 (d, *J* = 4.4 Hz, 1H), 6.76 (d, *J* = 7.5 Hz, 1H), 6.69 (d, *J* = 6.4 Hz, 1H). ^13^C NMR (126 MHz, CDCl_3_) *δ* 171.83, 171.83, 171.61, 171.61, 158.02, 148.71, 148.37, 147.88, 147.40, 142.14, 141.60, 141.21, 139.23, 138.95, 137.86, 137.60, 136.97, 136.17, 133.52, 132.64, 130.40, 129.47, 129.05, 128.96, 128.91, 128.70, 128.51, 128.27, 127.91, 127.87, 127.62, 123.92, 123.28, 123.23, 120.67. HRMS calculated for C_42_H_28_N_4_ M + H^+^ 624.2378, found: 625.2387.

## Conflicts of interest

There are no conflicts to declare

## Supplementary Material

RA-009-C9RA02875G-s001
