# Supplementary material for: A thermally activated delayed fluorescence exciplex to achieve highly efficient and stable blue and green phosphorescent organic light-emitting diodes
Source: RSC Adv. 2019 Jul 31;9(41):23810–7. doi: 10.1039/c9ra02875g (PMC9069484; doi:10.1039/c9ra02875g)

Electronic Supplementary Material (ESI) for RSC Advances.
This journal is © The Royal Society of Chemistry 2019

Supporting information

Thermally Activated Delayed Fluorescence Exciplex to Achieve Highly Efficient and Stable Blue and Green Phosphorescent Organic Light-Emitting Diodes

Dehai Dou,[a,b‡] Peng Wu,[a‡] Zhangcheng Liao,[a,b] Jian Hao,[b] Jianhua Zhang,[a] Zixing Wang [a*]

[a] Key Laboratory of Advanced Display and System Applications, Ministry of Education, Shanghai University, Shanghai, 200072, China.

[b] Department of Chemistry, Shanghai University, 99 Shangda Rd, Shanghai, 200444, China.

Corresponding Author

*E-mail: zxwang78@shu.edu.cn

‡These authors equally contributed to this work.

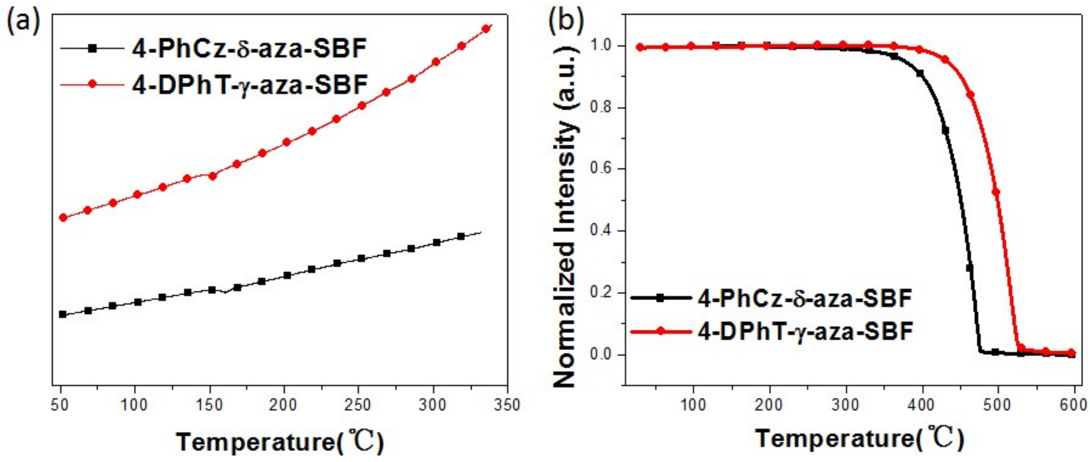

Figure S1. (a)DSC curves of **4-PhCz-*δ*-aza-SBF** and **4-DPhT-*γ*-aza-SBF**. (b) TGA curves of **4-PhCz-*δ*-aza-SBF** and **4-DPhT-*γ*-aza-SBF**.

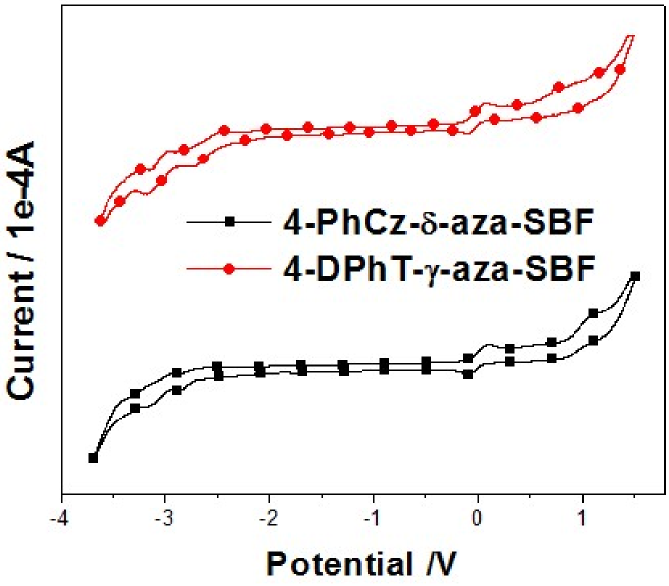

Figure S2. Cyclic voltammograms curves of **4-PhCz-*δ*-aza-SBF** and **4-DPhT-*γ*-aza-SBF** in DMF solution.

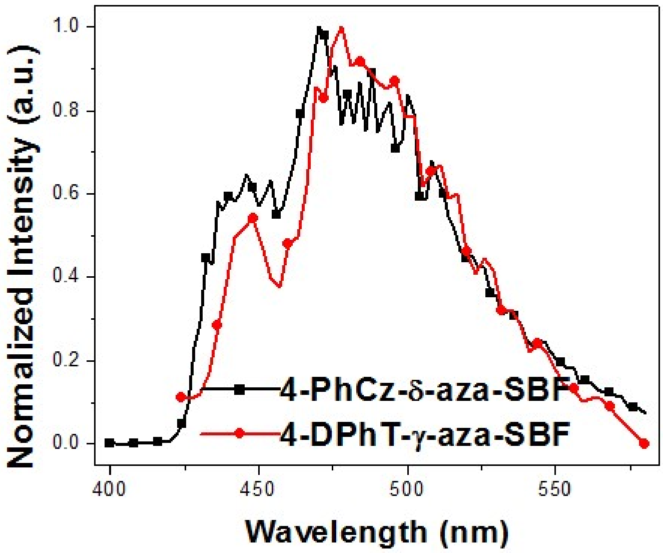

Figure S3. PL spectra of **4-PhCz-*δ*-aza-SBF** and **4-DPhT-*γ*-aza-SBF** at 77 K

^{1}H NMR and ^{13}C NMR

Compound **4-PhCz-*δ*-aza-SBF**

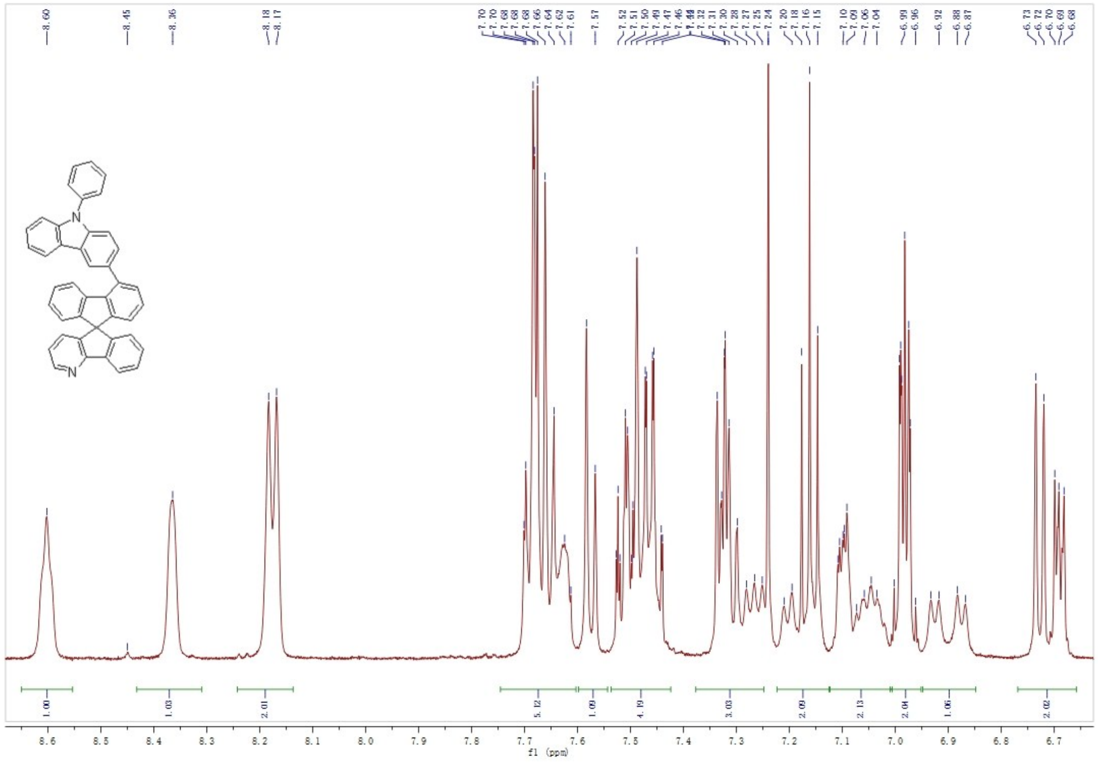

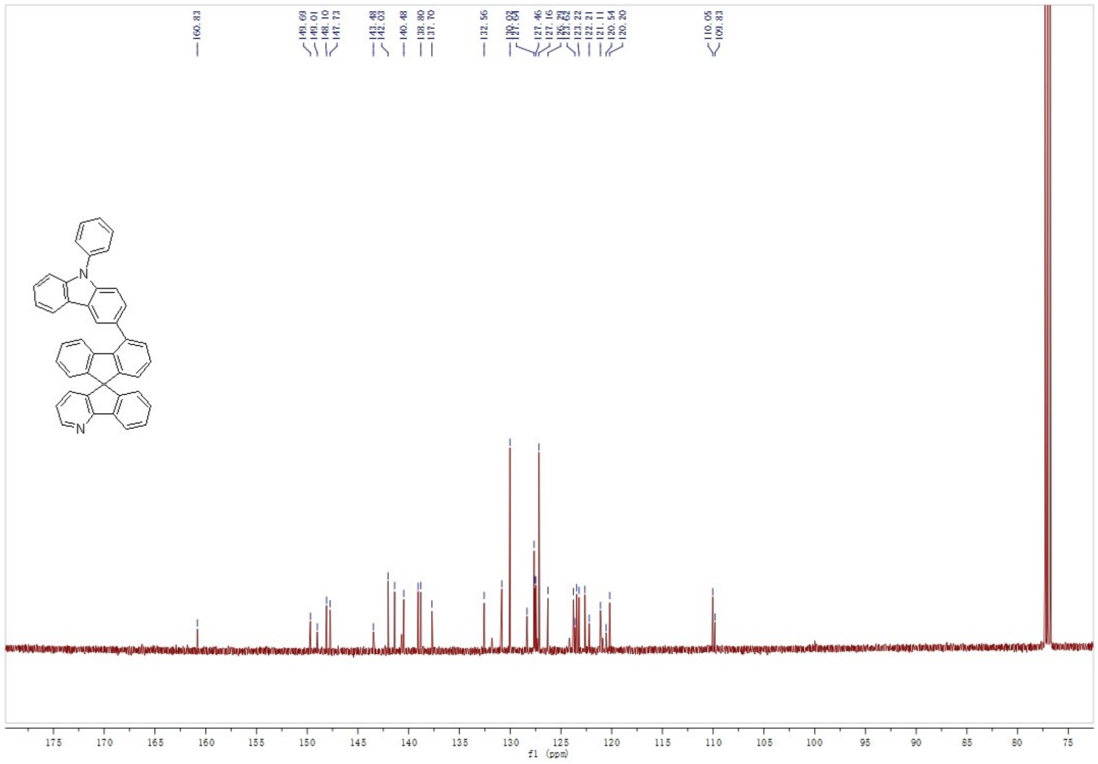

Compound **4-DPhT-*γ*-aza-SBF**

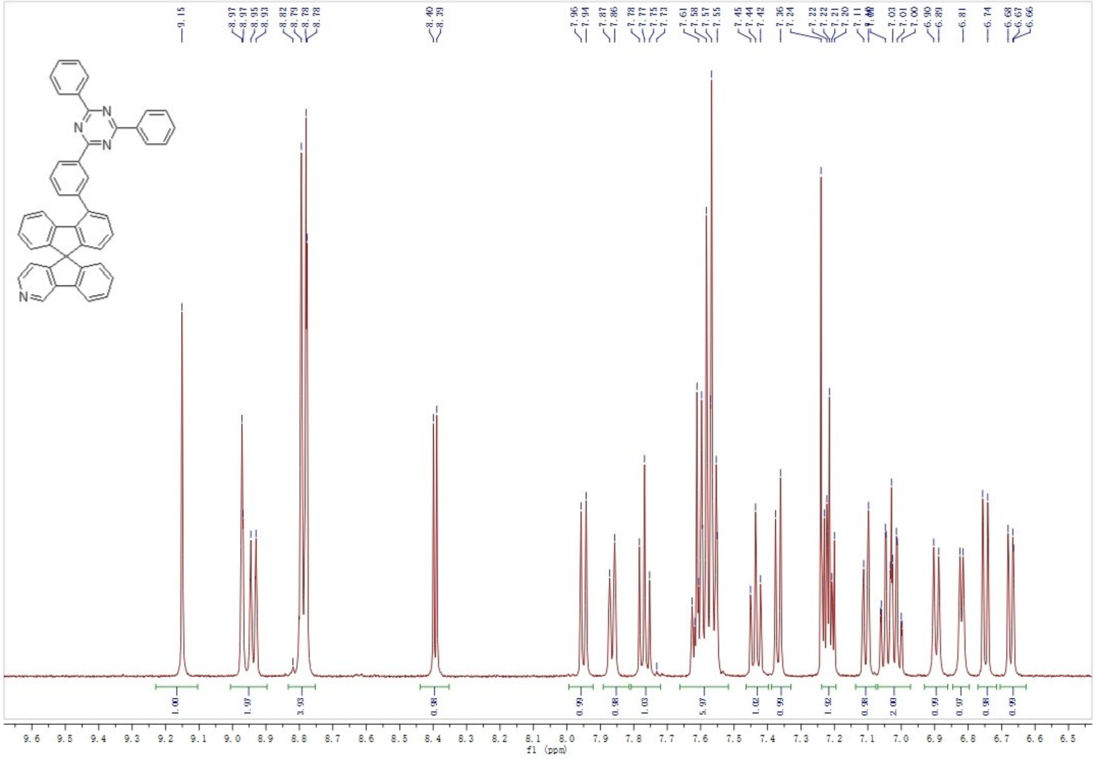

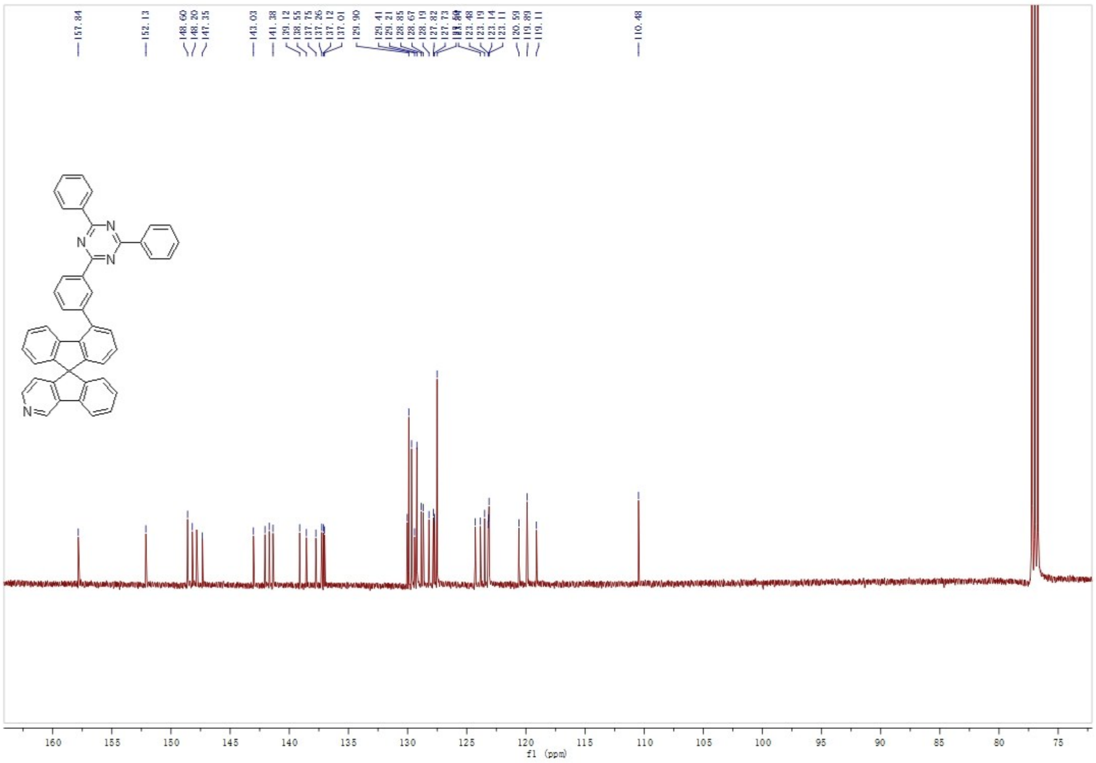

Supplement: RA-009-C9RA02875G-s001 [file RA-009-C9RA02875G-s001.pdf]
